# Lessons from Ethiopian coffee landscapes for global conservation in a post-wild world

**DOI:** 10.1038/s42003-024-06381-5

**Published:** 2024-06-10

**Authors:** Kristoffer Hylander, Sileshi Nemomissa, Joern Fischer, Beyene Zewdie, Biruk Ayalew, Ayco J. M. Tack

**Affiliations:** 1 Department of Ecology, Environment and Plant Sciences, Stockholm University, 106 91 Stockholm, Sweden; 2 Department of Plant Biology and Biodiversity Management, Addis Ababa University, Addis Ababa, Ethiopia; 3 Leuphana University, Faculty of Sustainability, Scharnhorststrasse 1, 21335 Lueneburg, Germany

**Keywords:** Conservation biology, Agroecology

## Abstract

The reality for conservation of biodiversity across our planet is that all ecosystems are modified by humans in some way or another. Thus, biodiversity conservation needs to be implemented in multifunctional landscapes. In this paper we use a fascinating coffee-dominated landscape in southwest Ethiopia as our lens to derive general lessons for biodiversity conservation in a post-wild world. Considering a hierarchy of scales from genes to multi-species interactions and social-ecological system contexts, we focus on (i) threats to the genetic diversity of crop wild relatives, (ii) the mechanisms behind trade-offs between biodiversity and agricultural yields, (iii) underexplored species interactions suppressing pest and disease levels, (iv) how the interactions of climate change and land-use change sometimes provide opportunities for restoration, and finally, (v) how to work closely with stakeholders to identify scenarios for sustainable development. The story on how the ecology and evolution of coffee within its indigenous distribution shape biodiversity conservation from genes to social-ecological systems can inspire us to view other landscapes with fresh eyes. The ubiquitous presence of human-nature interactions demands proactive, creative solutions to foster biodiversity conservation not only in remote protected areas but across entire landscapes inhabited by people.

## Abstract (web summary)

This perspective discusses five conservation challenges using a landscape in Ethiopia as the lens, exploring a story on how the ecology and evolution of coffee shape biodiversity conservation can inspire to view other landscapes with fresh eyes.

## Introduction

Most landscapes in the world, especially in more productive areas, are strongly modified by humans^1,2^ and can thus be denoted post-wild. This creates severe threats to biodiversity^3^. However, even in strongly modified landscapes there is often a substantial amount of heterogeneity that could sustain biodiversity^4^, and in some landscapes with a long history of human land use, active management is even considered a necessity to maintain the preferred biodiversity ^5,6^. Irrespective of the land-use history of a particular human-modified landscape, strategies for biodiversity conservation inevitably need to harmonize multiple, sometimes competing goals^7^. Conservation can be especially challenging when the management for multifunctionality also includes targets related to local or regional food security^8^. Given these realities, there is an urgent need to improve biodiversity conservation across the vast range of post-wild landscapes that now dominate our world^3^.

The coffee-dominated landscape in southwestern Ethiopia provides an interesting example of a post-wild landscape with high conservation values and strong human-nature interactions^9^. This region is representative of the indigenous distribution of Arabica coffee (*Coffea arabica*), but its wild roots, in terms of both genetics and ecology, are gradually getting lost due to climate change, deforestation and management intensification (Box 1 and lesson 1)^10^. Additional interesting features of the Ethiopian coffee landscapes are that they capture a wide management gradient from coffee growing essentially “wild” to intensively managed plantations of domesticated coffee; that growing and drinking coffee have been culturally important for centuries; and that coffee is a key component of the local and national economy (Box 1)^11,12^.

Drawing on this fascinating coffee-dominated landscape as our lens, we outline general challenges for biodiversity conservation in a post-wild world. Considering a hierarchy of scales from genes to multi-species interactions and social-ecological system contexts, we discuss (i) threats to the genetic diversity of crop wild relatives, (ii) the mechanisms behind trade-offs between biodiversity and agricultural yields, (iii) underexplored species interactions suppressing pest and disease levels, (iv) how climate change and land-use change together sometimes provide new opportunities for restoration, and finally, (v) integrative approaches with stakeholders to identify scenarios for sustainable development.

Box 1 The origin and history of Arabica coffee and its current management in Ethiopia Arabica coffee (*Coffea arabica* L.) is one of few species that almost everyone in the world has heard of. Yet, many aspects of its evolution and ecology are still to be explored. The genus *Coffea* in the Rubiaceae family has 124 species, with the most species rich countries being Madagascar and Tanzania^113^. The beans from three species are used by people for preparing the beverage we call coffee: *Coffea liberica* (rarely), *Coffea canephora* (most often traded as robusta c. 44 % of global market) and finally *Coffea arabica*, also called Arabica coffee or highland coffee, which is the most traded one (56%) and also considered to have the highest quality^114,115^. *C. arabica* differs from all other *Coffea* species by being an allotetraploid, with a double chromosome set originating from its hybrid origin^18^. The mother species are assumed to be *C. canephora* and *C. eugenioides*, which nowadays occur wild in for example Congo, Uganda and Kenya^18^. The hybridization event is estimated to have occurred >500,000 years ago^116^, but some authors suggest it is perhaps much more recent^18^.

Wild (or semi-wild) *C. arabica* still occurs as a small understory tree (but hereafter referred to as a shrub to avoid confusion with the trees forming the shading canopy above the coffee) in Afromontane evergreen forests of W, SW and SE Ethiopia, but also in small amounts in S Sudan (Boma plateau) and most likely in N Kenya (Mt Marsabit)^19,85,117^. No one knows how long coffee has been used in Ethiopia exactly, but it has traditionally been used both to drink and eat in various ways^11^. Linnaeus named it ‘arabica’ since it was known from Yemen. However, in Yemen it is grown with irrigation and had come from Ethiopia over the Red Sea^21^. In the late 15^th^ century and early 16^th^ century it was brought from Yemen to India and Réunion and further to Indonesia and the Americas^21^. Now the five countries with the largest coffee production (of the arabica bean) are: Brazil, Colombia, Ethiopia, Honduras, and Peru producing 41.0, 14.3, 7.35, 6.5 and 4.5 million 60 kg bags of coffee, respectively^118^.

In the Ethiopian highlands, coffee is a common plant as long as the climatic conditions are suitable, in short: not too cold, not too hot and not too dry^11,85^. It is estimated that the livelihoods of ~15 million people rely on different parts of the coffee value chain^111^. However, the production systems could be roughly divided into 4 or 5 typical systems^11,55^ that often occur in a mosaic in the same landscape. The first system is called (i) ‘forest coffee’. Here coffee is harvested from the forest understory with no or little management of the canopy cover or the coffee shrubs themselves. This coffee is sometimes called ‘wild coffee’ and marketed as such^19^. However, in many forests at the right altitude the understory is totally dominated by coffee and other wild shrubs seem to be strongly held back. It is thus likely that continuous but minor management over the years also in the forest coffee system has created the composition that is seen today^119^. A common smallholder system is called (ii) ‘semi-forest coffee’. Here coffee is grown under a canopy of indigenous trees, often in forest fragments embedded in a matrix of annual crop fields or in the edges of larger forests. If the sites are managed more intensively they are sometimes denoted as (iii) ‘semi-plantation coffee’^55^. The coffee shrubs are generally allowed to become old and large in both the semi-forest and semi-plantation system and the management intensity varies between sites. Another common system is called (iv) ‘garden coffee’ whereby coffee is grown intermixed with root crops, enset also known as false banana (*Ensete ventricosum*) or fruit trees. The shade trees can be both indigenous trees and fruit trees such as mango or avocado. Finally, large scale (v) ‘coffee plantations’ are quite rare and are characterized by a sparse cover of shade trees of only few species. In these large company-owned coffee plantations the coffee is regularly pruned, improved varieties (‘cultivars’) are used and chemical fertilizers and herbicides are regularly applied. Although it is possible to classify coffee cultivation into these five different systems, in many landscapes it is also perfectly reasonable to view it as a gradient in management intensity from no or little managed forest coffee to heavily managed plantation coffee^12,33^ (Figure). The variability in complexity of the shade cover from simple to complex is also described from Latin American coffee systems^50^. Yet, a key difference in the Ethiopian context is that the coffee is an indigenous plant from the moist Afromontane forests and hence that it is very difficult to truly differentiate between a natural forest with coffee and a forest modified by coffee management.

Figure: Ethiopian coffee systems. (a) Gradient in management intensity of Arabica coffee in many southwest Ethiopian landscapes showing how canopy cover as well as structural complexity of woody species is changing along the gradient. Within southwest Ethiopia, the different management systems coexist within a mosaic landscape. Note that the garden coffee system, which is common in some areas in Ethiopia, is not depicted here, but can be seen as an intensive management system. (b) The Ethiopian coffee systems house a rich biodiversity.
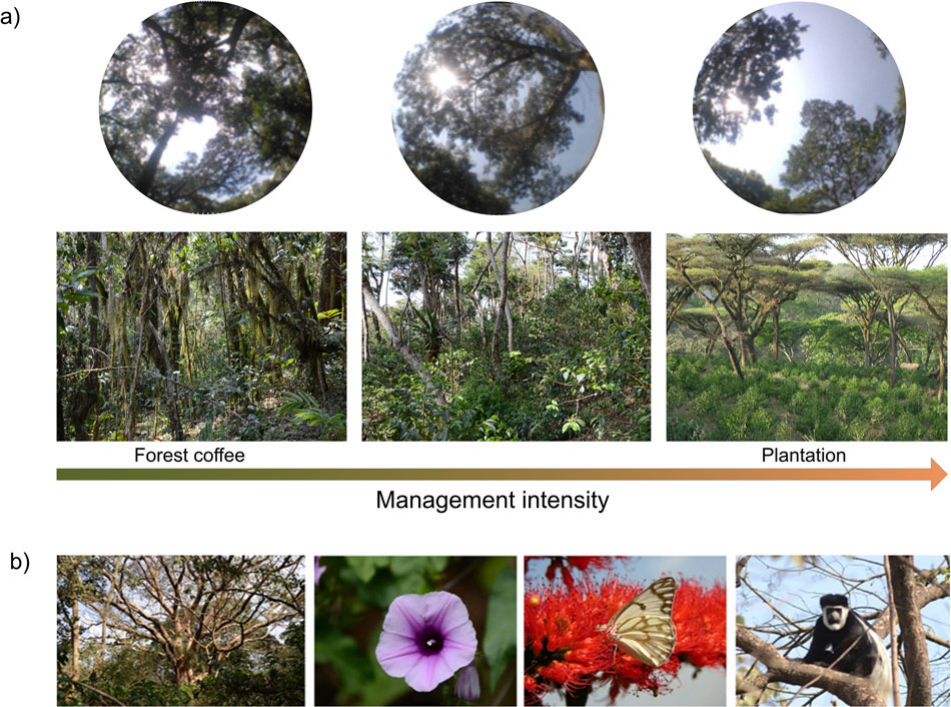

## Lesson 1: Safeguard natural selection processes in large areas to conserve genetic diversity of wild relatives of domesticated species

Populations of wild relatives of domesticated species are seen as extremely valuable to conserve, not least because their genetic variability provides a treasure trove for future breeding programs of their respective domesticated species^13,14^. It has been estimated that for crops alone, there are 58 000 species of wild relatives, indicating that conserving genetic variation in wild relatives of domesticated species is important in many areas across the world^15^. Where domesticated species and their wild relatives co-occur, introgression of genes into wild relatives from genetically related domesticated variants is a potentially major threat to genetic diversity^16,17^. Such co-occurrence of domesticated coffee and its wild relatives is common in Ethiopia – hence, an analysis of this situation can shed light on conservation challenges and solutions of crop wild relatives and more broadly on genetic diversity of domesticated species.

The coffee populations in the forests of Ethiopia have been identified as valuable because they contain Arabica coffee’s largest genetic variation^18^, but to what extent these populations are truly wild is questionable (Box 1). Local selection by farmers over centuries has produced locally adapted landraces in different parts of Ethiopia that are also seen as valuable in their own right^19^. Besides “wild” coffee and landraces, the Ethiopian coffee landscape also contains modern cultivars released from breeding programs starting in the 1970s aimed to combat coffee berry disease^19^. Different coffee berry disease-resistant cultivars now dominate the larger commercial coffee farms, but are also popular among smallholder farmers and are therefore intermixed in many places with coffee of other origins^20^.

**Fig. 1:**
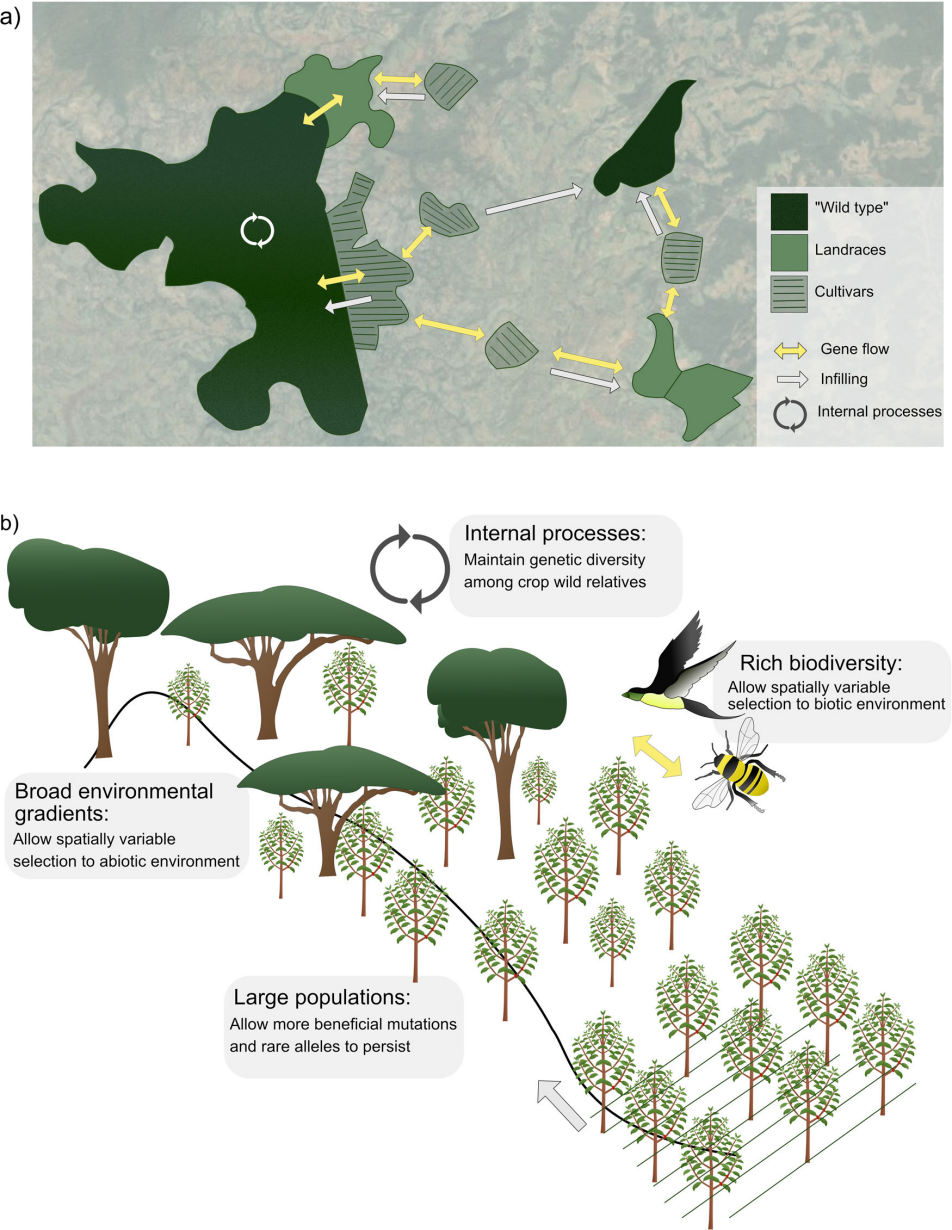
Challenges and opportunities in the conservation of genetic variation in crop wild relatives. **a** A conceptual figure showing gene flow and introgression of genes from domesticated variants into the wild, across a mosaic landscape with different types of coffee populations in southwest Ethiopia. Both landraces and cultivars are considered domesticated variants of the crop, but in fact also the “wild” coffee is often managed and thus exposed to human selection. Wild coffee, landraces and cultivars often occur in a spatial mosaic as a result of both current and historical management. Dispersal of seeds and pollen causes gene flow (yellow arrows), but also infilling from more intensively managed coffee farms into the forests (white arrows) directly affect the genetic composition. Since these processes have been ongoing for some decades, the spatial configuration of genetic composition is likely more mixed than this simplified conceptual figure implies. Internal processes are explained in the next panel. **b** If possible, conserving large populations under more or less natural conditions along abiotic and biotic environmental gradients would allow spatially heterogeneous and balancing selection to operate in crop wild relatives and reduce threats from genetic swamping and introgression from domesticated variants. Such measures would maintain genetic diversity and resilience to future environmental changes. The grey arrow denotes different kinds of gene flow from domesticated variants into the natural habitats.

Even though the genetic variability in Arabica coffee is low compared to many crop wild relatives^18^, Ethiopian coffee has a wider genetic base than coffee grown elsewhere in the world (Box 1)^19,21^. Moreover, the spatial variation in genetic composition across Ethiopian landscapes is complex, reflecting a coffee management history involving processes such as isolated traditional selection, trade, spread of coffee into reforested areas after depopulation due to wars, and current management intensification and spread of modern cultivars^20,22^. Thus, the valuable “wild” coffee gene pool is embedded within the broader context of a post-wild landscape and it is impossible to say what is truly “wild” coffee (Fig. 1a).

International literature on the protection of genetic variability of crop wild relatives often highlights two major threats^23^. One threat is that the genetic composition of the wild species will change due to the introgression of novel genes from domesticated variants, with associated changes in life-history and interactions. Commonly cited examples include introgression from genetically modified crops into weeds that become more difficult to control^24^. The second and related threat is that the domesticated variant is so common and the wild so rare that it leads to demographic and genetic swamping of the wild population and eventually extinction of genetic variation or even the wild taxon as such^16,25^. In the Ethiopian context, the risk of introgression of genes from coffee berry disease-resistant cultivars into the wild coffee gene pool has been highlighted (Fig. 1a)^26,27^. In principle, actions that protect wild populations from gene flow from domesticated variants would be effective. However, in the Ethiopian case, introgression of genes from landraces as well as from improved cultivars into the less managed coffee in the forests is likely unavoidable, given the need of farmers to ensure a decent productivity as well as the mosaic nature of the genetic composition of coffee across the landscape (Fig. 1a). Moreover, coffee is pollinated primarily by the honey bee (both wild and semi-domesticated)^28^, which is known to fly long distances and thus contributes to gene flow. The risks of genetic swamping of the genetically most diverse populations increase if the “wild” populations become very small in relation to the domestic populations^16^. Ethiopia is on such a trajectory since larger forests with little managed coffee are becoming more scarce and are increasingly being converted to more intensively managed coffee farms that use cultivars^29,30^.

The Ethiopian case challenges us to broaden our perspectives and reconsider our conservation toolbox, since fully isolating the wild coffee gene pool from the domesticated gene pool is likely unrealistic, and even the description of the problem as a matter of wild versus domesticated gene pools is overly simplistic (see Box 1). Because less intensively managed forest coffee populations are getting replaced by more intensively managed coffee plantations using improved cultivars, genetic swamping might be a larger threat to the genetic variation of Ethiopian coffee than from introgression per se from cultivated coffee outside the forests into the forest coffee. On this basis, the best and also most feasible way to ensure the long-term conservation of genetic variability of coffee is to maintain large areas of low management intensity across environmental gradients – even though this will also be challenging considering pressures on land as well as for higher yields and revenues. In these large areas, natural processes that maintain high genetic variation, such as spatially heterogenous selection and gene flow, can continue to operate (Fig. 1b)^31,32^. With such an approach, the risk of losing genetic variation through demographic and environmental stochasticity is reduced. Moreover, the risk of losing genetic diversity of wild coffee through introgression of genes from domesticated populations would be reduced also, as it is unlikely that introgressed genes will be selected for across multiple environmental gradients (see also e.g. ref. 21). As long as such large areas exist, the need to put a ban on the use of improved varieties outside the forests in the same landscape will be of lower priority. Such a ban would be extremely difficult to implement since coffee berry disease is considered the largest challenge for farmers in this region, with few alternative management strategies existing^33^.

To protect native habitats and allow wild populations to evolve under natural conditions with associated biodiversity should be an important strategy also for other wild relatives of domestic species^34^. For example, wild relatives to cacao (*Theobroma cacao* L.) occur in Colombian forests, of which a large proportion is not protected^35^. However, in other cases, such as the European wild apple, there might be too few, small and fragmented wild populations to rely solely on protecting wild populations and enhancing natural processes^25^. It is also important to acknowledge the challenges and possible downsides of a strict land sparing approach in multifunctional landscapes, where people depend on forest resources (see e.g.^36^ and lesson 5). In the Ethiopian case, we suggest that it would still be possible for people to extract resources from the forests as long as the area available is large, a rich biodiversity is maintained and coffee populations are subjected to natural processes of dispersal, pollination, and pest control (Fig. 1b). In other settings, for other wild relatives of domesticated species, additional or different conservation approaches might be needed, depending on the traits of the species^24^ and the status of the local habitats^25^. Finally, we suggest that the value of genetic variation in wild relatives of domesticated species, e.g. for future breeding programs, could add an important argument for conservation of native habitats where these species grow, as is the case in Ethiopia.

Notwithstanding the importance of the wild relatives of domesticated crops, maintaining genetic variation of the domesticated crops themselves is an important target in itself, including in landscapes far from their origin^37^. In the case of Arabica coffee, low genetic variation combined with rapid evolution of diseases, such as coffee rust, have been shown to be detrimental, for example in Latin America^38^. Thus, the diversity of landraces and their genetic variation are also worthy of conservation^39^. This can be achieved, for example, by continuing traditional selection and promotion of varieties from different villages, as is the case, for example, in the speciality coffee market.

## Lesson 2: Explore the mechanisms underpinning relationships between management, biodiversity and yield to maintain both provisioning ecosystem services and biodiversity

When exploring the relationship between biodiversity and provisioning ecosystem services, such as food or fibre, it is common – but far from universal (see below) – to find a trade-off between the two^40–42^. Especially in a context of single-crop oriented industrial agriculture, it is difficult to increase the production of goods without harming the biodiversity or ecosystem^43^. Given the many negative effects, such as increased nutrient loads and habitat simplification, on ecosystems from intensive agriculture^44^, it is important to understand the mechanisms behind trade-offs between yield and biodiversity, in order to provide robust advice for developing more sustainable alternatives^45^. A first thing to acknowledge is that the relationships between yield and biodiversity are seldomly consistently negative or linear along wider gradients of yield and biodiversity, because different management activities often affect yield and biodiversity components differently^46^. Enhanced knowledge about these relationships can open up windows of opportunities for management choices that enhance biodiversity while keeping yields at reasonable levels, or that improve yields in low-yielding systems without losing biodiversity. This is demonstrated by a recent meta-analysis that shows that diversification of agriculture in many cases actually can improve biodiversity without compromising yield, but that the outcomes are context dependent^47^. Agroforestry systems are often highlighted as systems that can go some way to alleviate the strength of the trade-off, and sometimes can even provide win-win solutions for conservation and provisioning ecosystem services, especially when other ecosystem services are taken into account^42,48–51^. In this context it is important to stress that a farmer often is more interested in overall benefits from an agroecosystem than in the yield of the major crop per se. This increases the opportunities for win-win solutions in cases where parts of the agroecosystem (e.g. shade trees) provide multiple types of benefits. The Ethiopian case, with its unique broad gradient of management intensity, gives us some new insights into problems and opportunities along yield-biodiversity trade-off gradients.

**Fig. 2:**
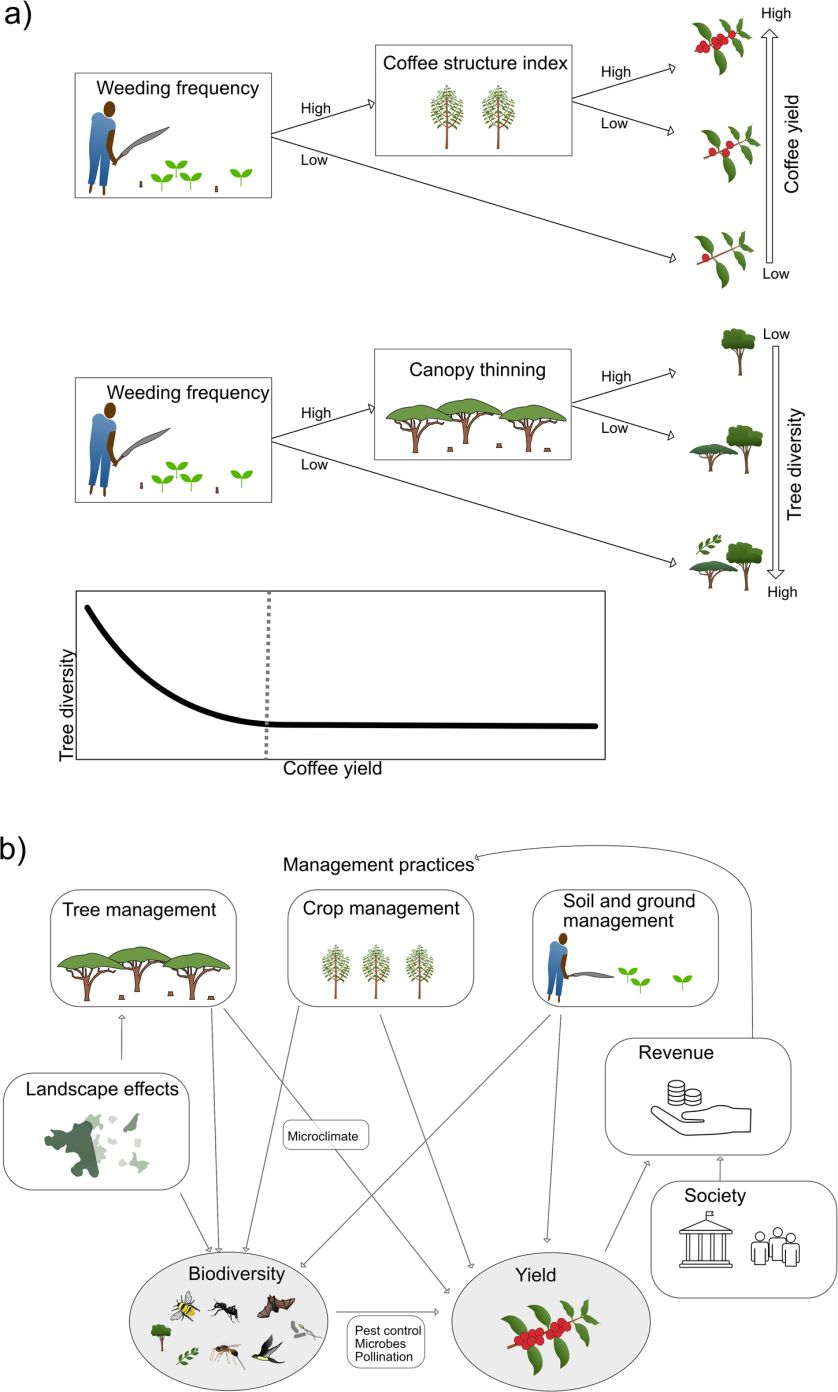
Examining the trade-off between biodiversity and provisioning ecosystem services. **a** In shade-coffee systems in southwest Ethiopia we identified three major management variables related to different types of intensification activities: Canopy cover (reflecting thinning of shade trees as well as a selection of trees with a canopy that is more permeable), Coffee Structure Index (capturing the active pruning of the coffee shrubs, but also correlated to the use of improved cultivars), and Weeding frequency (slashing of the ground vegetation, but also strongly correlated to other activities at the ground such as fertilization). **a** top and middle: When separately modelling the drivers of yield and tree species richness using the statistical approach ‘random forest’, we found that the same management action (high weeding frequency) explained both the lowest yields and the highest biodiversity. Yet, for the set of sites with higher yields and lower biodiversity, we found that variation in yield and biodiversity, respectively, were driven by different types of management: higher yields were attained by pruning (and associated practices), while higher biodiversity was associated with less canopy thinning. **a** Bottom: Trade-off curve between biodiversity and coffee yield (modified from^12^). In the left part of the curve, we have the situation where the same management driver affects both the yield and the biodiversity, and in the right part of the curve, we have the situation where different drivers affect yields and biodiversity. **b** (i) In order to understand yield-biodiversity relationships it is important to separately study the effect of different management activities on yields and biodiversity. The figures show a conceptual framework describing how different components of management in an agroforestry system can affect both yields and biodiversity, as well as how external factors such as landscape connectivity and social-ecological contexts (affecting for example the cost of labour) interact to shape the relationships between yield and biodiversity. Biodiversity can also directly influence yields and thus modify the direct effects of management on yield.

The Ethiopian semi-forest production system has been suggested to be among the most bird-friendly and mammal-friendly agroforestry systems in the world^52,53^. However, despite the complex three-dimensional structure of a shade grown coffee farm, such sites generally have a depauperate biodiversity compared to little managed forest sites, with for example fewer specialized forest birds, lianas, and orchids^54–56^. A recent study along a broad management gradient from forest coffee to plantation coffee explored the drivers of yield and biodiversity and the shape of the resulting trade-off (Fig. 2a). When separately modelling the drivers of yield and biodiversity, we found that the same management action (weeding frequency) explained both the lowest yields and the highest biodiversity (Fig. 2a top & middle). This implies that when people start to intensify coffee cultivation in low yield-high biodiversity systems, there will be a drastic drop in biodiversity values (left part of the curve in Fig. 2a bottom). Yet, for the set of sites with intermediate to high yields, we found that yield and biodiversity were driven by different types of management: higher yields are attained by pruning (and associated practices), while higher biodiversity is associated with higher canopy cover (Fig. 2a top & middle). Thus, in much of the landscape outside near-natural forests, there may be opportunities for improving yields without losing biodiversity, or for improving biodiversity without losing yields (right part of the curve in Fig. 2a bottom).

As shown in the Ethiopian case, it is important to tease apart the mechanisms behind yield-biodiversity relationships. In the context of agroforestry systems, we suggest that there are three main management interventions that could each affect both yield and biodiversity, likely in different ways: managing the canopy, the crop plant itself and the ground layer/soil (Fig. 2b). It is also important to study different aspects of biodiversity, since for example tree diversity is directly linked to the management of the shade trees, while for example soil microbes might respond more to management actions applied at the ground layer^57^. Besides trees that shape the shade, microclimate and to some degree soil and water properties, other biotic interactions such as pests, pest-controlling organisms and pollinators impact yields^46^ (see also lesson 3 below). Finally, farmers might primarily not be interested in only yields but in a sustainable income – which underlines the importance of understanding the social-ecological context to better understand opportunities and challenges for attaining both conservation of biodiversity and sustainable livelihoods (see lesson 5 below for additional details). The conceptual framework in Fig. 2b can help researchers to move from a simplified view of a negative, linear trade-off between yield and biodiversity to explore context-specific opportunities to reach several societal goals in the landscape (depicted for agroforestry systems in Fig. 2b). Yet, even in agroforestry systems like shade grown coffee or cocoa, it is naïve to believe that it is possible to manage for maximum productivity and revenue at the same time as maintaining the highest biodiversity values^40,58^, as also exemplified in the Ethiopian case. However, by exploring the underlying drivers of biodiversity-yield trade-off curves along broad gradients it is easier to develop conservation and management policy and plans both at the landscape scale and site level to reach societal goals of both yields (revenue) and biodiversity^59^.

### Lesson 3: Leverage beneficial biodiversity-crop interactions

Intensive agriculture is one of the largest threats to biodiversity worldwide^44^. Not only the use of various pesticides, but probably even more the simplification of the landscapes is detrimental to biodiversity^60^. Many ecologists thus argue that we need a paradigm shift that utilizes ecosystem services of wild biodiversity to reach the dual goals of both food security and biodiversity conservation^61,62^. From a farmer’s perspective, it is valuable to manage the system to increase positive interactions such as pollination, while decreasing negative interactions such as attacks by pests and diseases^63–66^. Yet, it is not easy to attain such knowledge because many positive interactions are cryptic and involve small and inconspicuous species^67,68^. The Ethiopian case is interesting in the sense that it is a quite biodiversity rich system largely managed without pesticides and fungicides. The example highlights that we have barely started to understand the role of the many interactions. To illustrate the level of complexity involved, we give three examples of largely hidden beneficial biodiversity-crop interactions that might partly explain why coffee farmers in southwestern Ethiopia at present are not very concerned about several globally-devastating foliar pests and diseases^69^.

**Fig. 3:**
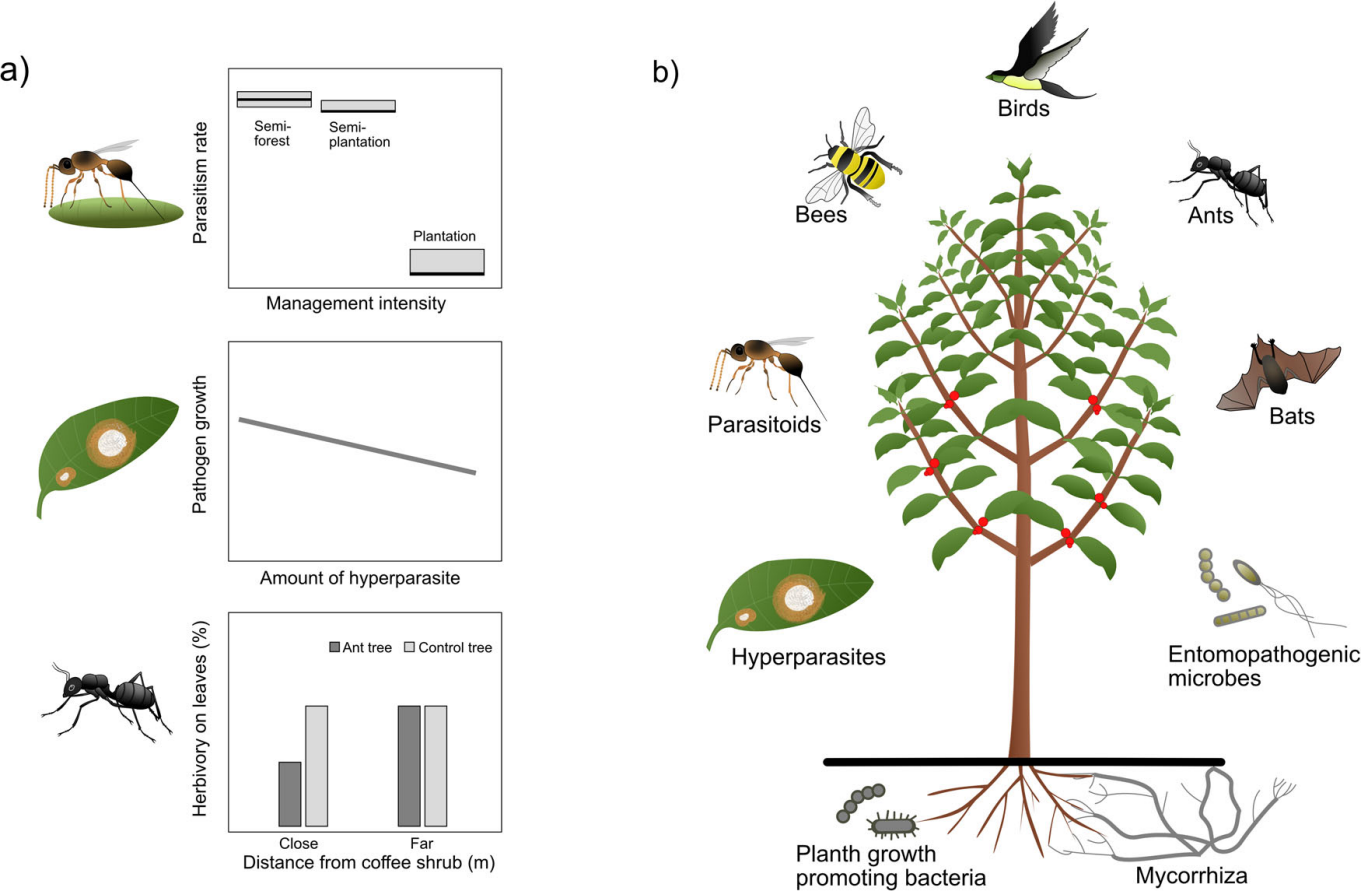
Beneficial and underexplored biodiversity in crop systems. **a** Three examples of cryptic top-down control agents on coffee pests in southwest Ethiopian coffee systems. Top: The parasitism rate of the coffee blotch miner is higher in semi-forest coffee types than in more intensively managed plantations^71^. Middle: A hyperparasite (white) supresses the growth of coffee leaf rust (orange) from the rainy to the dry season^73^. Bottom: Herbivory is lower on coffee shrubs below shade trees with nests of *Crematogaster* ants^75^. **b** Globally, there are many underexplored beneficial interactions: There are many studies on birds predating on herbivores and on pollination by bees, fewer on interactions with ants and mycorrhizal fungi, and very few on more cryptic interactions for example from parasitoids, hyperparasites, bats and beneficial microbes, let alone studies on multiple interactions or three-way interactions.

The first example considers the role of parasitoid wasps (Fig. 3a: top). Although coffee has many secondary substances in its tissues, presumably to counteract herbivores, pathogens and other negative interactions, it is still attacked by various species of herbivores including both specialists and generalists^70,71^. For example, several specialized moths attack the coffee leaves, of which the coffee blotch miner *Leucoptera caffeina* is the most abundant^72^. Parasitoid wasps, however, lay eggs in or on the larvae of the blotch miner and thereby possibly reduce the risk of outbreaks of this pest. Interestingly, the proportion of parasitized blotch miner larvae was found to be higher in complex smallholder farmers’ sites than in more simplified plantation coffee sites (Fig. 3a: top^71^). While less often studied, pathogen species have—just like pest species—a wide range of natural enemies. The most common fungal disease on coffee in southwest Ethiopia is coffee leaf rust caused by *Hemileia vastatrix*. As a second example of a little known beneficial species, it seems like the hyperparasitic fungus *Lecanicillium lecanii* can hold back the growth of coffee leaf rust (Fig. 3a: middle^73^). A final example underlines the important role of ants, which are often abundant in tropical systems^74^. In Ethiopia, arboreal *Crematogaster* ants are seen as an annoyance by coffee berry pickers, but actually provide a service of supressing herbivory levels on coffee leaves (Fig. 3a: bottom^75^).

These are three examples of beneficial biodiversity in the Ethiopian coffee landscape that are rather cryptic and little known, which together with other still unknown interactions might have immense value for sustainable management practices, and which hitherto neither farmers nor plantation managers are aware of (cf.^76^). However, our understanding of the Ethiopian system is still very limited. Specifically, we do not know to what extent the depicted interactions affect average yields or the risks of outbreaks of the different focal pests or diseases. We have indications from interviews with farmers that coffee rust and leaf miners at present do not cause massive defoliation in the Ethiopian systems^73^ as has been reported from more intensively managed areas outside of the indigenous distribution of coffee (e.g. in Latin America^77^). However, yields are quite low in many of the Ethiopian farms and to what extent it is possible to increase the yield without losing biodiversity is still an open question (see lesson 2).

In general, the extent to which it is possible to offset yield losses (or rather revenue/income losses, see lesson 5) through biodiversity-friendly agriculture is much debated, and certainly context dependent^60,76^. However, the biodiversity crisis challenges us to further explore the possibilities to develop practices to sustainably harvest provisioning ecosystem services by utilizing the pest and disease regulating ecosystem services from a diverse biodiversity. Thus, in terms of general lessons, the Ethiopian coffee case illustrates that there is a tremendous but hopefully rewarding task ahead for both basic and applied ecologists to explore species interactions within natural and agricultural settings, and identify which factors shape the strength of pest and disease regulation (Fig. 3b)^78^. It could be particularly timely to take advantage of next generation sequencing to identify the important role that inconspicuous microbes, both below and above ground, play in regulating pests and diseases. When we also unravel the drivers of the beneficial organisms, we can manage the landscape in such a way that it enhances the regulating role of natural enemies in suppressing pests and diseases^79^, which is also referred to as conservation biocontrol^80^. The area of origin of the crop might also sustain a wealth of natural enemies or plant beneficiaries that have co-evolved with the pests and diseases, and sometimes these can be introduced into other areas for pest and disease control, which is referred to as classic biocontrol. For landscapes with crop wild relatives, where co-evolution is likely, it is therefore important to conserve reference areas where beneficial species interactions can continue to evolve and be studied (see also lesson 1).

### Lesson 4: Identify not only risks for conservation but also novel opportunities for restoration arising from the interaction of climate and land-use change

It can be difficult to disentangle the causes of declining biodiversity because multiple, interacting drivers can change simultaneously^81,82^. Currently, populations of species, more or less across the whole globe, respond to the combined effects of climate change and land-use change^83,84^. The interaction of land use change and climate change generally worsens the threats to biodiversity and thus increases the challenges for conservation. However, in rare cases, upslope shifts in climate isoclines can open up new and unforeseen conservation or restoration opportunities. The Ethiopian case study illustrates this well. Here, climate change opens up new opportunities for restoration of indigenous trees in landscapes that have been completed deforested in the past, and are currently dominated by annual crop agriculture.

The ecology, management, and economic value of coffee make it a key driver of tree cover and biodiversity distribution within the highlands of southwest Ethiopia. Generally, coffee in Ethiopia is managed under a canopy of indigenous shade trees, even within intensively managed plantations. Because coffee is sensitive to both too warm and too cold temperatures, it is generally restricted to altitudes between 1200 and 2000 m a.s.l.^85^, and this has protected the tree cover in the mid-altitudes of the Ethiopian highlands during a general period of deforestation^30^.

**Fig. 4:**
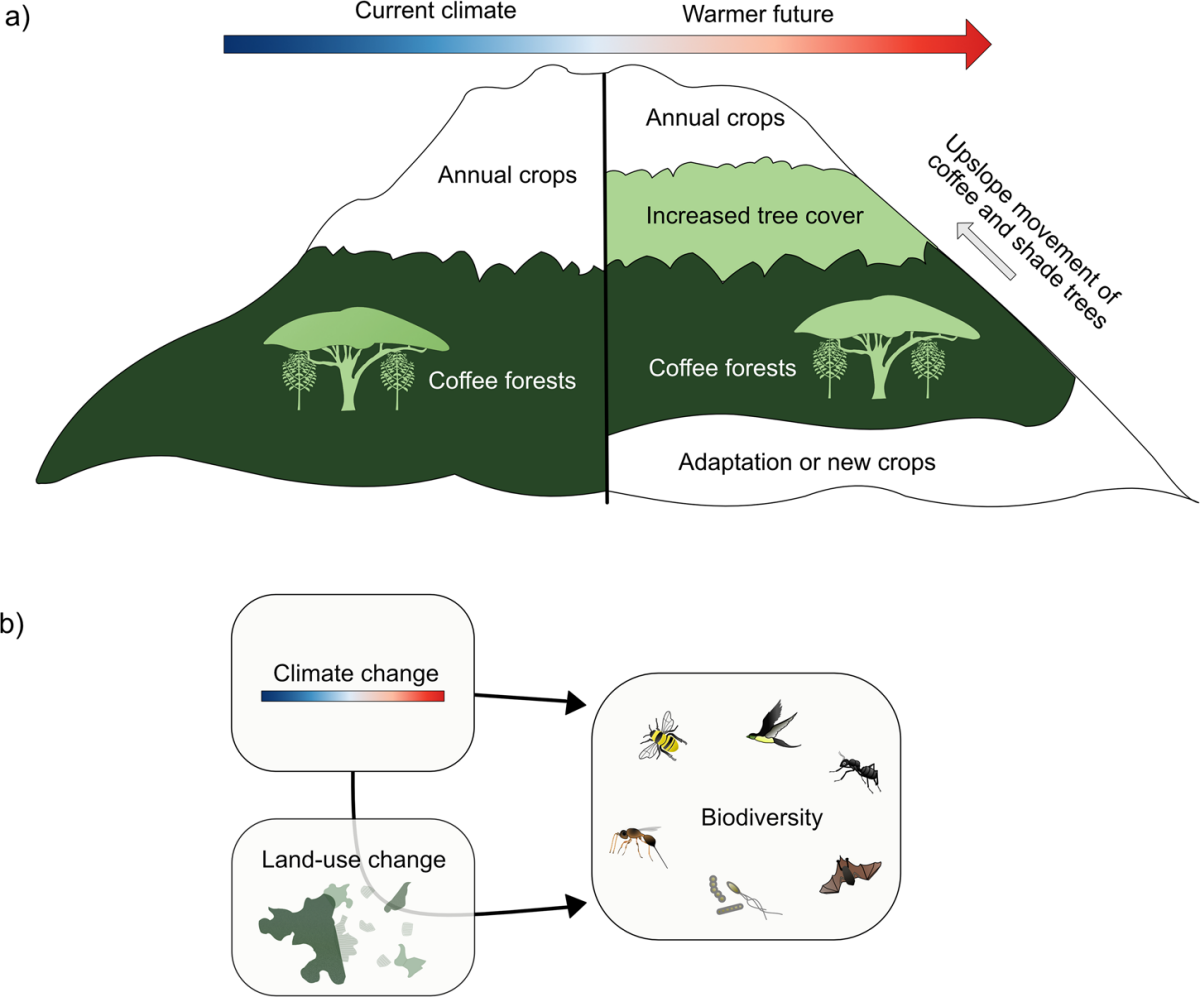
Despite climate change posing a key threat to biodiversity in general, its indirect effects could in some instances also create restoration opportunities. **a** In southwest Ethiopia, when the climate gets warmer farmers start to grow coffee at higher altitudes. This is likely to increase tree cover at higher altitudes, which had previously been cleared and currently is dominated by annual crop production. **b** In general, there are both direct and indirect effects of climate change on biodiversity. Indirect effects via land-use changes can pose severe risks to biodiversity, but need to be taken into account when planning adaptation policies to, if possible, search for new windows of restoration opportunities.

The climate of the coffee growing regions in Ethiopia^10^, as well as in many other countries (e.g.^86^), is predicted to change considerably, pushing its cultivation to higher altitudes, if available, or forcing farmers to implement more effective management of the understory microclimate^87,88^. In southwest Ethiopia, farmers have already started to plant coffee at higher altitudes than in the past, accompanied with planting shade trees or allowing them to regenerate naturally^66^^, personal observation^. This expansion of shade-coffee systems occurs in areas currently dominated by annual crops, which decades to centuries ago had expanded at the expense of Afromontane forests^30^. If native shade trees are promoted during the expansion of coffee cultivation into currently treeless areas at high altitudes, this could have positive consequences for forest biodiversity that has been pushed back in these landscapes (Fig. 4a). Preliminary data suggest that preferred trees are native trees with favourable traits such as nitrogen fixing legumes or fast-growing trees that can soon provide shade, even though sometimes exotic trees and fruit trees are also used (Tack, personal observation). Yet, with the same logic there is a risk of reduced tree cover at the lower altitudinal range limit in southwest Ethiopia if coffee farming needs to be abandoned because conditions get unsuitably hot. The changes taking place thus come with new opportunities at high altitudes, but also with new threats at low altitudes. At low altitudes, it is important to find solutions that protect the tree cover, perhaps by exploring possibilities for other shade-grown crops than coffee. In case coffee agroforestry is retained in these areas an increase in shade cover may mitigate some of the warming effects at the same time as improving biodiversity values^88^.

Even if the specific climate-land use interactions for coffee in southwest Ethiopia cannot be translated directly to other landscapes (but see^89^), the fact that indirect effects of climate change via land-use change can sometimes open up new opportunities for positive biodiversity outcomes provides food for thought. As stated above, we certainly do not want to downplay the great risks generally associated with combined climate and land-use change^90^. However, in some instances, unforeseen combined changes in climate and land-use might pave the way for novel conservation opportunities (Fig. 4b). Or put differently: there will be a need for climate adaptation, and that could sometimes be combined with restoration actions that might have been difficult to suggest in a static situation^91^. For example, after a storm that damage a monoculture tree plantation a more diverse forest could be established. What is also clear is that changes of many different kinds are inevitable and continuously need to be accommodated^91,92^.

## Lesson 5: Use a social-ecological systems perspective for conservation planning

In multifunctional landscapes, conservation challenges cannot be solved without understanding the socioeconomic context of local people^7,93^. Especially in smallholder farming landscapes in the tropics, local livelihoods are intimately intertwined with biodiversity^94^. The coffee landscapes of southwest Ethiopia demonstrate that a detailed understanding of social-ecological complexity can help to identify unsustainable trajectories for a given region, as well as identify concrete steps towards more successful outcomes for both people and biodiversity.

**Fig. 5:**
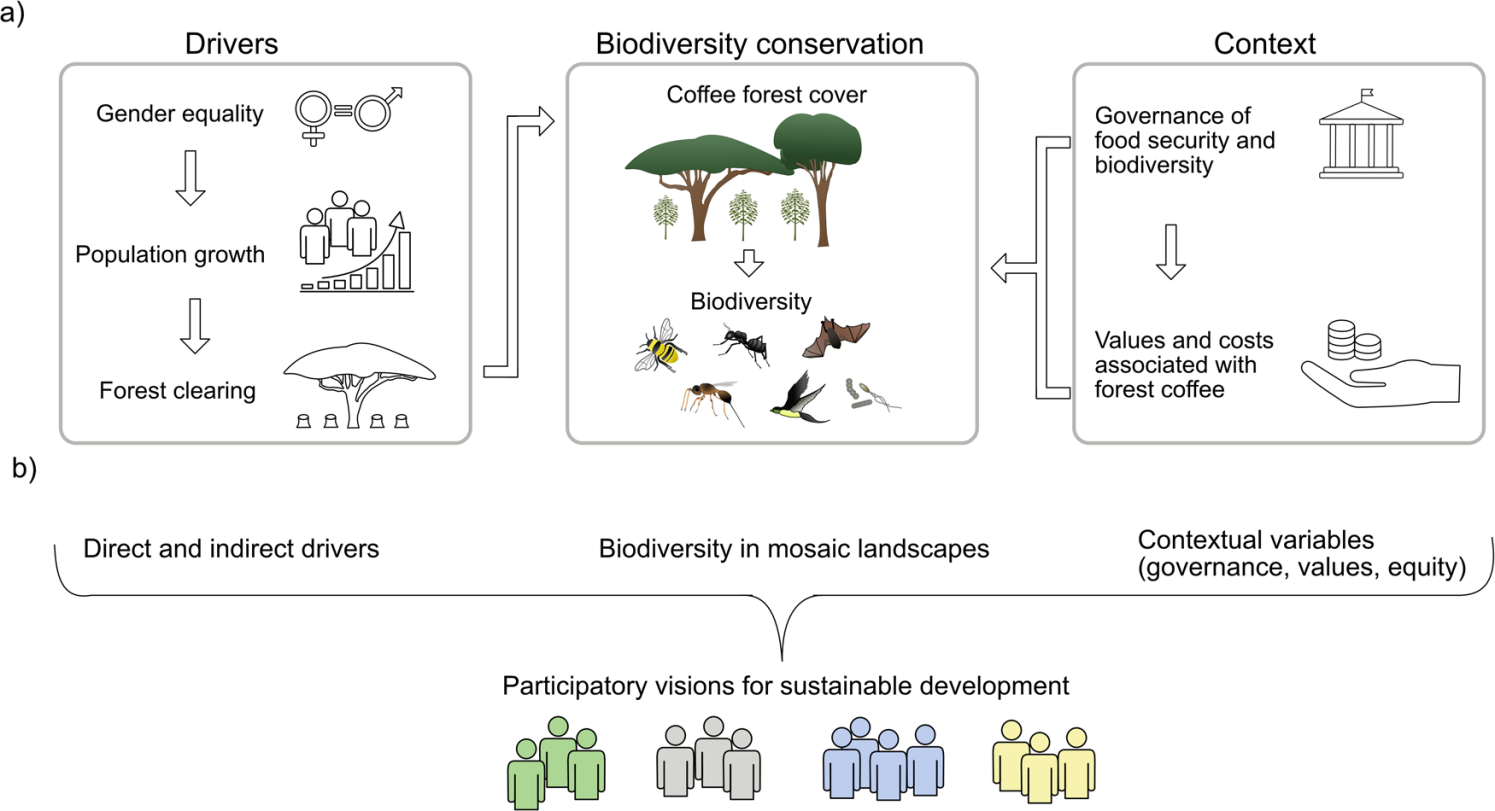
Social-ecological systems perspective for conservation planning. **a** In southwest Ethiopia spatial and temporal variation in rates of forest clearing can only be understood in the context of the interplay between population growth, top-down governance and how people value coffee both from a cultural and economic point of view. **b** In general, conservation planning needs to engage groups of stakeholders that together discuss how drivers and contextual variables affect conservation targets, in order to bring opportunities and challenges for more long-term and just conservation to the table.

Forest clearing by smallholder farmers has been the most important direct cause of biodiversity loss in southwest Ethiopia for many decades. The indirect cause underpinning this is the need to obtain new land for food production, which is critical because food security remains a challenge for a large proportion of households^95^ (Fig. 5a). The local population is still growing rapidly^96^, despite gradual improvements in gender equity^95^. To safeguard both food security and biodiversity conservation, some stakeholders envision conventional agricultural intensification, whereas others favour a focus on resilience and agroecological methods^97^. The push for intensification is also visible in the coffee sector, with the conversion of parts of native forest into plantations^98^.

To unpack the dynamics of land use, it is important to understand how stakeholders perceive the benefits and disbenefits of alternative land uses (Fig. 5a). Coffee is valued for its economic exchange value, but it also plays an important cultural role in the landscape^99^. Coffee is drunk by most households several times a day, and Ethiopia is known for its special coffee ceremony practiced on all festive occasions including if a neighbour is visiting^11^. Thus, growing coffee has a much more important role than being a sole cash crop, although it plays an important role for many households’ income as well. It is also important to understand that farmers are not managing their coffee for higher yields per se (see lesson 2), but as a complementary crop to food crops; because it is the combination of food crops and cash crops that provides the highest resilience to shocks, and hence the highest food security^95^. The traditional western view that higher yields are necessarily the most important goal thus ignores the important complementary contribution of resilience, in contexts where questions of expenses, labour, choice of buying or producing food and other considerations need to be constantly evaluated in order to construct viable livelihoods^100^. Moreover, although coffee forest is valued for both economic and cultural reasons, it also harbours numerous wildlife species that can destroy local food crops^101,102^. Thus, it could be favourable for a family to have a farm at some distance to the forest edge as long as it is still possible to access the forest for key resources^102^. Local land use decisions therefore take place in a context of both benefits and disbenefits associated with coffee forest, which play out differently depending on the location of a particular household in relation to forests and whether the climatic conditions in the forest are suitable for coffee or not. It would be short-sighted to assume that only economic incentives guide local land use (and conservation) decisions—relational and cultural values, considerations of risk spreading, as well as ecosystem disservices, will also influence different stakeholders’ visions of a sustainable landscape^103^. Importantly, only by understanding the social-ecological context can we predict future changes in the landscape, which is crucial if we, for example, want to take advantage of opportunities for conservation and restoration arising through dynamic processes such as climate change (as described in lesson 4).

In addition to alternative landscape trajectories being associated with different benefits and disbenefits in different parts of the landscape, these may also be borne by different stakeholders—plantation-grown export coffee, for example, benefits companies and remote consumers (arguably more so than local labourers), while locally traded forest coffee generates a mixture of modest economic benefits and cultural benefits for local people^104^. To successfully conserve coffee and its genetic diversity, as well as the high levels of biodiversity associated with these forests, it is central to understand how diverse actors together shape the governance and use of forests (Fig. 5a). In the Ethiopian coffee landscape context, both governance structures (e.g. rules) and processes (e.g. of decision-making) can pose challenges and opportunities for biodiversity conservation^105^. For ecologists, it is important to be aware of this complexity – for example, top-down decision-making could easily overlook the needs of less influential community members (such as poor households or women); there can be large discrepancies between ideas generated at high levels of government versus local capacities to implement such ideas; and staff might fluctuate frequently creating a lack of institutional continuity and history^105^. This complexity requires ecologists to engage differently with decision-makers than simply through the publication of scientific outputs. One possibility is to work with stakeholders in transdisciplinary ways; that is, co-designing possible solutions through a combination of scientific and stakeholder inputs^106^. Based on transdisciplinary workshops with over 30 local stakeholder groups, multiple scenarios are plausible for how Ethiopia’s coffee landscape and its biodiversity may change over the next 20 years. One possible scenario is the creation of a new biosphere reserve^97^. Unlike scenarios associated with intensified coffee or food production, this scenario has a higher likelihood of generating equitable benefits for local people as well attracting international actors with an interest in sustainability (rather than economic returns only) to the study area^107^. By co-creating and identifying sustainable long-term trajectories that are beneficial for multiple stakeholders at both high and low governance levels can we as scientists, facilitate decision-making that is based on long-term visions rather than short-term gains.

In terms of general lessons, the Ethiopian case underlines that successful biodiversity conservation in a post-wild world is intimately intertwined with socioeconomic considerations^93,108^. Areas protected from intensive management are effective for biodiversity conservation in principle – but need to be complemented with other approaches since what happens in the remainder of the landscape is also vital^8,109^. To integrate conservation and socioeconomic concerns requires not only consideration of agricultural yields or profits, but also consideration of broader issues such as governance, local values and gender dynamics, and citizen participation (Fig. 5b). Most importantly, working with the pluralistic perspectives of multiple stakeholders in a given region will likely lead to more successful (and more just) conservation initiatives than the traditional method of imposing strictly protected areas^94^.

## Outlook

By zooming in on coffee in a landscape in its indigenous distribution, we extracted five conservation lessons that are likely to be relevant to many landscapes in our human-dominated world. Beyond these global lessons, also in its own right, the Ethiopian coffee landscape deserves global attention^9^ – most notably because it is the home of *Coffea arabica* with its genetic resources in increasingly sparse montane forests, embedded in a cultural context with a long history of utilization of coffee that still sustains the livelihoods of millions of people^19^. Even if the whole landscape could be characterized as a cultural landscape, rather than a mosaic of wild and managed patches, the long-term conservation in the least managed parts of the landscape is likely dependent on the connectivity across the whole agroecological landscape^110^. As such, biodiversity conservation is important along the entire management gradient present within the landscape (see Box 1).

The five lessons discussed above are clearly interlinked. In the Ethiopian case, for example, the coffee populations of largest genetic variation (lesson 1) are also threatened by climate change (lesson 4) in the sense that few natural forests occur at higher altitudes into which coffee populations could extend their ranges^111^. Another example is that we need more knowledge in the Ethiopian cases on to what extent beneficial biodiversity (lesson 3) contribute to maintaining productivity at intermediate levels of management (lesson 2), even if we know that the more or less untouched forests have very low coffee productivity despite high general biodiversity (of both beneficial species and other species).

Lesson 5 show that the social-ecological context is crucial to understand for all conservation implications, and for balancing what works best for a broad range of stakeholders in the long term—and not only to maximise short-term yields. Given this complexity, it might be questionable to what extent our lessons learned can be generalized beyond southwestern Ethiopia. Yet, we have tried to highlight aspects in all of the five reviewed themes that are worth exploring further in other landscapes across the globe where people live and interact with local ecosystems (Figs. 1–5). Most importantly, the challenges cannot be ignored, because the majority of the world’s landscapes are modified by humans and *de facto* managed for multifunctionality^3,4^. Accepting the ubiquitous presence of human impact and ongoing natural or anthropogenic changes in nature implies a need for active policies and management that could create a rich biodiversity not only in remote protected areas but across entire landscapes and regions^112^.

### Reporting summary

Further information on research design is available in the Nature Portfolio Reporting Summary linked to this article.

### Supplementary information

Reporting summary
